# Supplementary material for: Hippocampal place cells have goal-oriented vector fields during navigation
Source: Nature. 2022 Jul 6;607(7920):741–6. doi: 10.1038/s41586-022-04913-9 (PMC9329099; doi:10.1038/s41586-022-04913-9)
Supplement: Supplementary file 1 — This file contains Supplementary Figs. 1–10 and a full description for Supplementary Video 1. [file 41586_2022_4913_MOESM1_ESM.pdf]

nature portfolio

https://doi.org/10.1038/s41586-022-04913-9

Supplementary information

Hippocampal place cells have goal-oriented vector fields during navigation

In the format provided by the authors and unedited

Supplemental Information

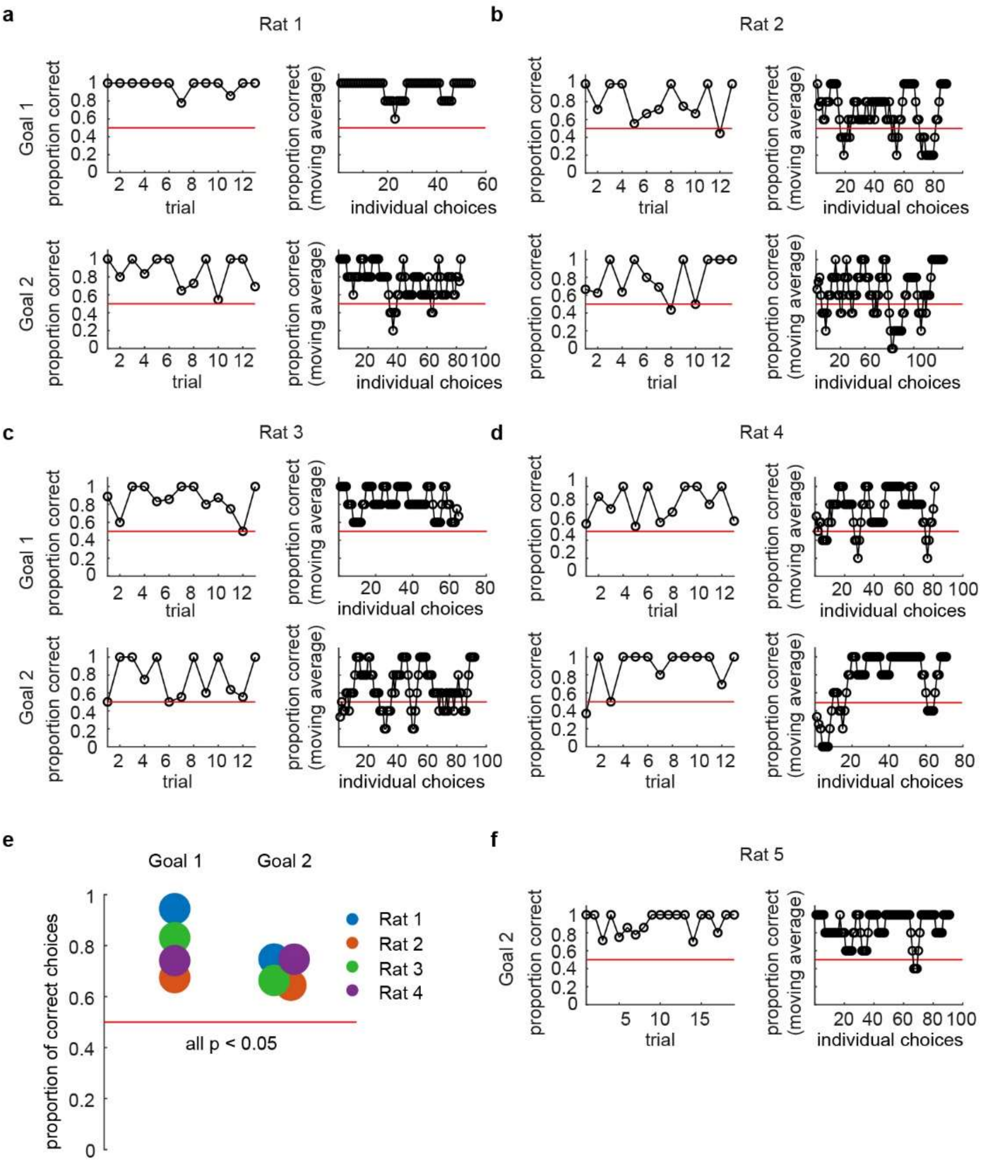

Supplemental Figure 1 │ Animals successfully navigated to new goal locations in the goal switch sessions. a, Top left, Proportion of choices that were correct to Goal 1 averaged by trial for rat 1. Top right, Running average of correct choices (averages of every 5 consecutive choices) to Goal 1 for rat 1. Bottom right and left, as at top, but to Goal 2. **b-d**, As in (**a**) for rats 2, 3, and 4, respectively. **e**, The total

proportion of correct choices for each rat for goals 1 and 2. Each rat made significantly more correct than incorrect choices for both goals; Rat 1, goal 1: $p = 2.92 \times 10^{-12}$, goal 2: $p = 7.51 \times 10^{-6}$; Rat 2, goal 1: $p = 0.0013$, goal 2: $p = 0.0042$; Rat 3, goal 1: $p = 6.03 \times 10^{-8}$, goal 2: $p = 0.0023$; Rat 4, goal 1: $p = 1.69 \times 10^{-5}$, goal 2: $p = 3.88 \times 10^{-5}$; two-sided binomial test within each animal. **f**, Rat 5's behavioural performance after a week of training to Goal 2. Left, Proportion of choices that were correct to Goal 2 averaged by trial. Right, Running average of correct choices (averages of every 5 consecutive choices) to Goal 2.

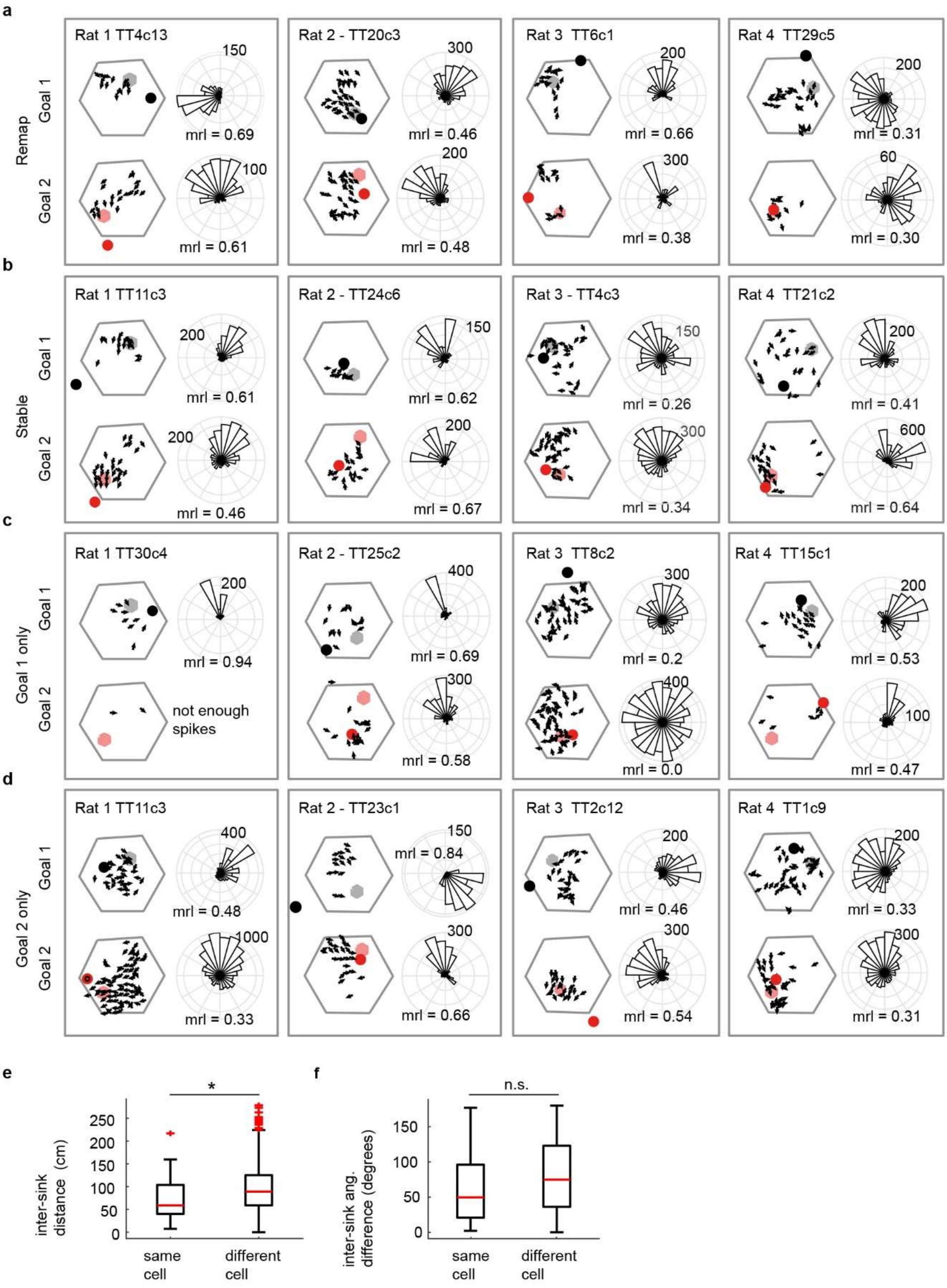

Supplemental Figure 2 │ Examples of ConSink cells recorded during the goal switch sessions. a, Example cells that were significantly modulated during both Goal 1 and Goal 2 epochs, but whose ConSink changed position. Vector fields (left)

depict mean head direction at binned spatial locations. The ConSink and goal location depicted in black (Goal 1) or red (Goal 2). Right, Polar plot showing the distribution of head directions relative to the ConSink. **b**, As in (**a**), but examples whose ConSink positions did not change. **c**, Examples of cells that were only significantly tuned during Goal 1. Where sufficient spikes were fired (min. 500), the best candidate ConSink position for Goal 2 is plotted. **d**, As in (**c**), but examples that were only tuned during Goal 2. **e**, Distances between Goal 1 and Goal 2 sinks within cells that had sinks during both epochs (e.g. the cells shown in (**a**) and (**b**)) were smaller than those between all other possible pairs of Goal 1 and Goal 2 ConSink cells. Wilcoxon rank sum test, two-sided, $n = 28$ cells (same cell) and 3024 cell pairs (different cell) from 4 animals, $z = -2.41$, $p = 0.016$. **f**, Differences in mean direction between Goal 1 and Goal 2 sinks within cells that had sinks during both epochs (e.g. the cells shown in (**a**) and (**b**)) were smaller than those between all other possible pairs of Goal 1 and Goal 2 ConSink cells, but this difference was not significant (Wilcoxon rank sum test, two-sided, $n = 28$ cells (same cell) and 3024 cell pairs (different cell) from 4 animals, $z = -1.49$, $p = 0.14$). Together, (**e**) and (**f**) suggest that sink position may not be solely dependent on the goal. For box plots in (**e**) and (**f**), the central mark indicates the median, and the bottom and top edges of the box indicate the 25th and 75th percentiles, respectively; the whiskers extend to the most extreme data points within 1.5 times the interquartile range away from the bottom or top of the box, and all more extreme points are plotted individually using the '+' symbol.

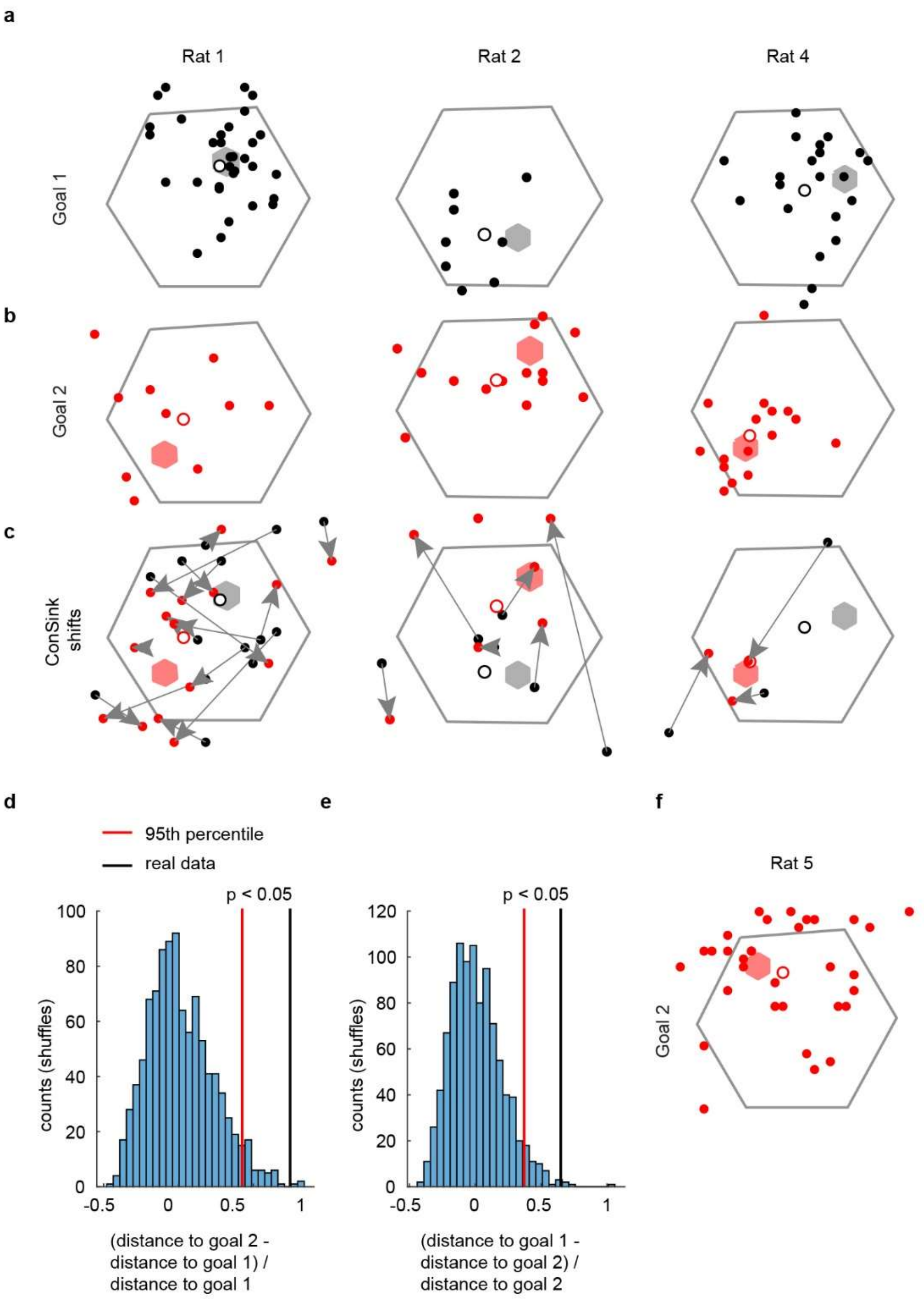

Supplemental Figure 3 │ ConSinks move towards the new goal in all recorded animals. a, Spatial distribution of ConSinks active only in Goal 1 (grey hexagon) before the goal switch. Average ConSinks, open circles. **b**, Spatial distribution of ConSinks active only in Goal 2 (red hexagon) after goal switch. **c,** Arrows show movement of ConSinks from Goal 1 to Goal 2. **d**, During the Goal 1 epoch, the difference in sink distance to Goal 2 relative to Goal 1 (normalized by distance to

Goal 1) was greater than for random pairs of platforms (Monte Carlo simulation, single-sided, $p < 0.05$). **e**, Similarly, during the Goal 2 epoch, the difference in sink distance to Goal 1 relative to Goal 2 (normalized by distance to Goal 2) was also greater than for random pairs of platforms (Monte Carlo simulation, single-sided, $p < 0.05$). **f**, Spatial distribution of ConSinks in Rat 5 after multiple days of training to Goal 2 (red hexagon).

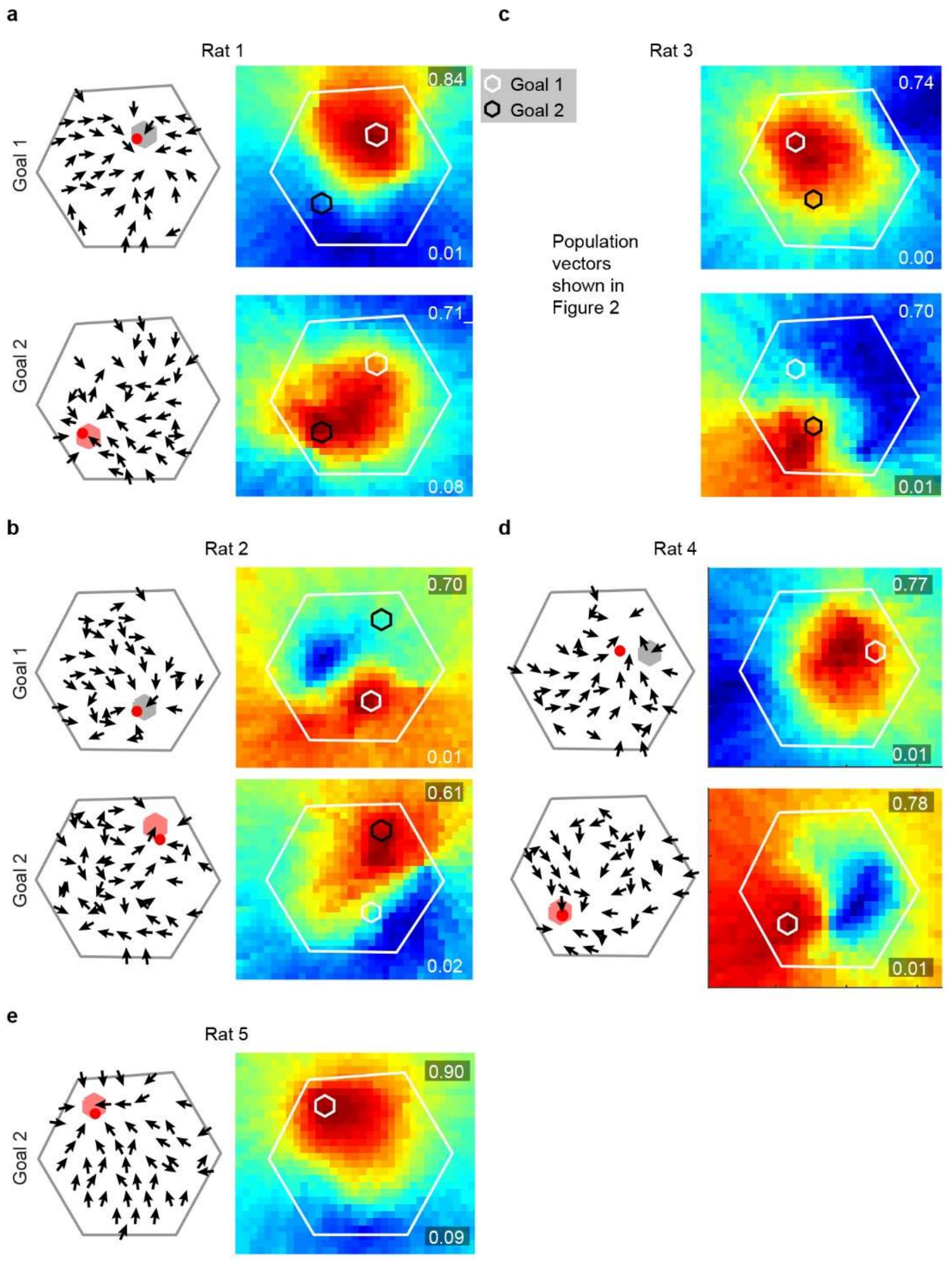

Supplemental Figure 4 │ MRL maps calculated from population vector fields show stronger tuning to the current goal than the subsequent or previous goal. a, Population vector fields for Rat 1 (left), and MRL maps (right) calculated from the population vector fields during Goal 1 (top) and goal 2 (bottom; these maps are analogous to the single cell MRL map shown in Extended Data Figure 5b). The MRL values shown in Fig. 2i are taken from the goal-centred locations in these maps. **b-d**, as in (a) but for rats 2-4, respectively (note that rat 3's vector fields are

shown in Figure 2g and h). **e**, Population vector fields for Rat 5 (left), and MRL maps (right) recorded after multiple days training to Goal 2.

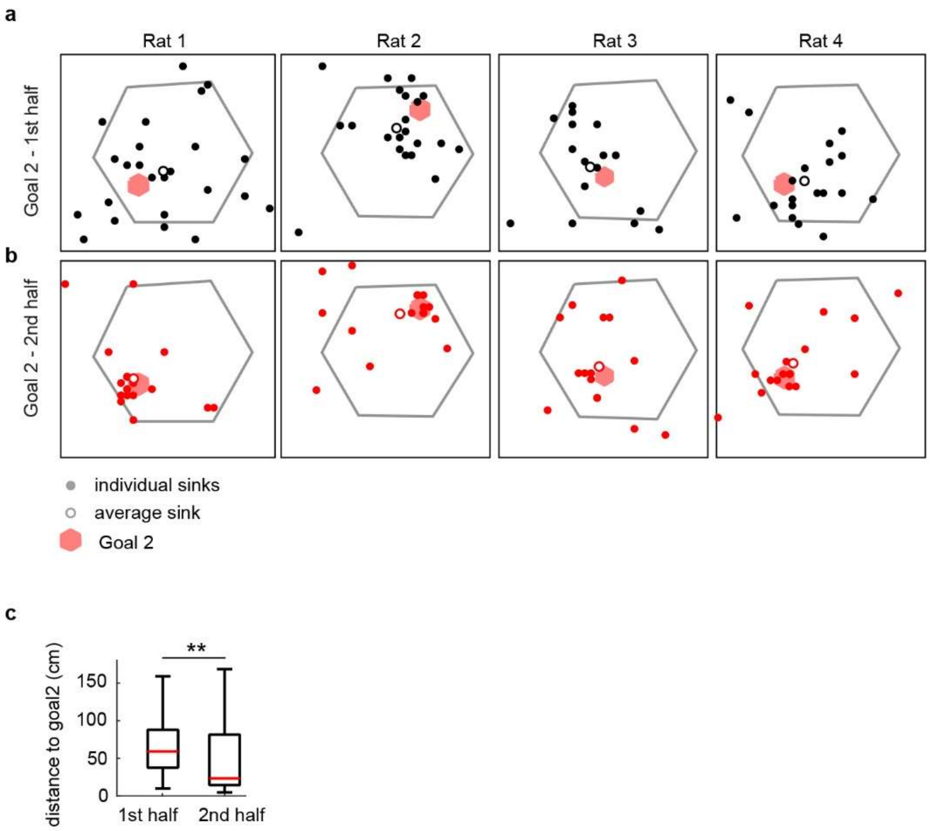

Supplemental Figure 5 │ Average ConSinks move towards the new goal with additional trials. Spatial distribution of ConSinks during the first (**a**) and second (**b**) halves of Goal 2 training for each rat. Average ConSinks, open circles. **c**, ConSinks moved closer to the new goal in the second half of training (Wilcoxson sign rank test, two-sided, n = 80 cells from 4 animals (note that 1 cell was excluded due to insufficient number of spikes in 1 of the 2 halves), $z = 3.35$, $p = 1.90 \times 10^{-4}$. For box plots, the central mark indicates the median, and the bottom and top edges of the box indicate the 25th and 75th percentiles, respectively; the whiskers extend to the most extreme data points away from the bottom or top of the box).

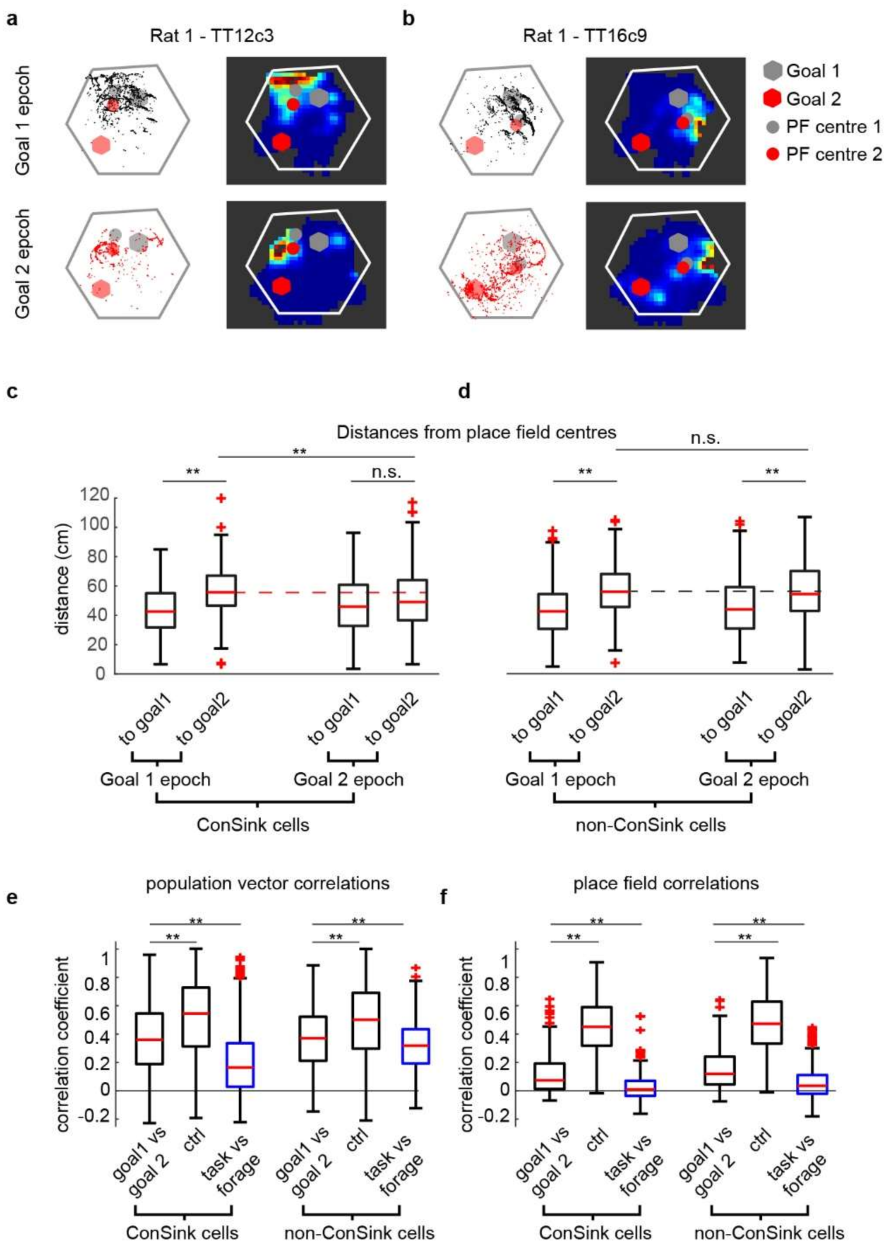

Supplemental Figure 6 │ Place fields do not show clustering around goal 2 after the goal switch. a, Spike plots and rate maps from a representative ConSink cell during Goal 1 and Goal 2 epochs. Note that the rate map centre of mass (place field centre) shifts slightly towards Goal 2 after the goal switch, but remains closer to Goal 1. **b**, A second representative example cell. Again, centre of mass shifts slightly towards Goal 2 but remains closer to Goal 1. **c**, **d**, Summary of place field centre – goal distance data. Note that results are similar for both ConSink (**c**) and non-

ConSink (**d**) cells; place field centres are initially closer to Goal 1 than to Goal 2, but, only in ConSink cells, they move towards Goal 2 after the goal switch. Wilcoxon rank sum test, two-sided, ConSink cells Goal 1 epoch, n = 157 cells from 4 animals, $p = 2.65 \times 10^{-7}$; ConSink cells Goal 2 epoch, n = 155 cells from 4 animals, p = 0.095; ConSink cells Goal 1 epoch – goal2 vs Goal 2 epoch – goal 2, $p = 9.82 \times 10^{-4}$; non-ConSink cells Goal 1 epoch, n = 174 cells from 4 animals, $p = 2.30 \times 10^{-8}$; non-ConSink cells Goal 2 epoch, n = 193 cells from 4 animals, $p = 3.13 \times 10^{-5}$; non-ConSink cells Goal 1 epoch – goal2 vs Goal 2 epoch – goal 2, p = 0.79. No corrections made for multiple comparisons. **e**, **f**, Remapping in both ConSink (**e**) and non-ConSink (**f**) cells after the goal switch is less than is observed after the switch from honeycomb task to open field foraging. Remapping defined as significantly lower correlation than within control data; control data consists of session 1 (i.e. static goal) data split into first and second halves. Wilcoxon rank sum test, two-sided, population vector correlations: ConSink cells goal 1 vs goal 2, n = 1299 population vectors from 4 animals; ctrl, n = 2541 population vectors from 4 animals ; task vs forage, n = 1697 population vectors from 4 animals. "goal1 vs goal2" vs ctrl, $p = 1.31 \times 10^{-56}$; "goal1 vs goal2" vs "task vs forage", $p = 2.23 \times 10^{-79}$; non-ConSink cells goal 1 vs goal 2, n = 1303 population vectors from 4 animals; ctrl, n = 2549 population vectors from 4 animals; task vs forage, n = 2062 population vectors from 4 animals; "goal1 vs goal2" vs ctrl, $p = 8.29 \times 10^{-47}$; "goal1 vs goal2" vs "task vs forage", $p = 7.19 \times 10^{-12}$; place field correlations, ConSink cells goal 1 vs goal 2, n = 162 cells from 4 animals; ctrl, n = 324 cells (each cell repeated twice, once from goal 1 and once from goal 2) from 4 animals; task vs forage, n = 143 cells from 4 animals. "goal1 vs goal2" vs ctrl, $p = 9.46 \times 10^{-48}$; "goal1 vs goal2" vs "task vs forage", $p = 1.40 \times 10^{-9}$; non-ConSink cells goal 1 vs goal 2, n = 199 cells from 4 animals; ctrl, n =

398 cells (each cell repeated twice, once from goal 1 and once from goal 2) from 4 animals; task vs forage, n = 309 cells from 4 animals. “goal1 vs goal2” vs ctrl, $p = 1.22 \times 10^{-54}$; “goal1 vs goal2” vs “task vs forage”, $p = 1.17 \times 10^{-14}$. No corrections made for multiple comparisons. ** indicates $p < 0.001$. For box plots in (**c**-**f**), the central mark indicates the median, and the bottom and top edges of the box indicate the 25th and 75th percentiles, respectively; the whiskers extend to the most extreme data points within 1.5 times the interquartile range away from the bottom or top of the box, and all more extreme points are plotted individually using the '+' symbol.

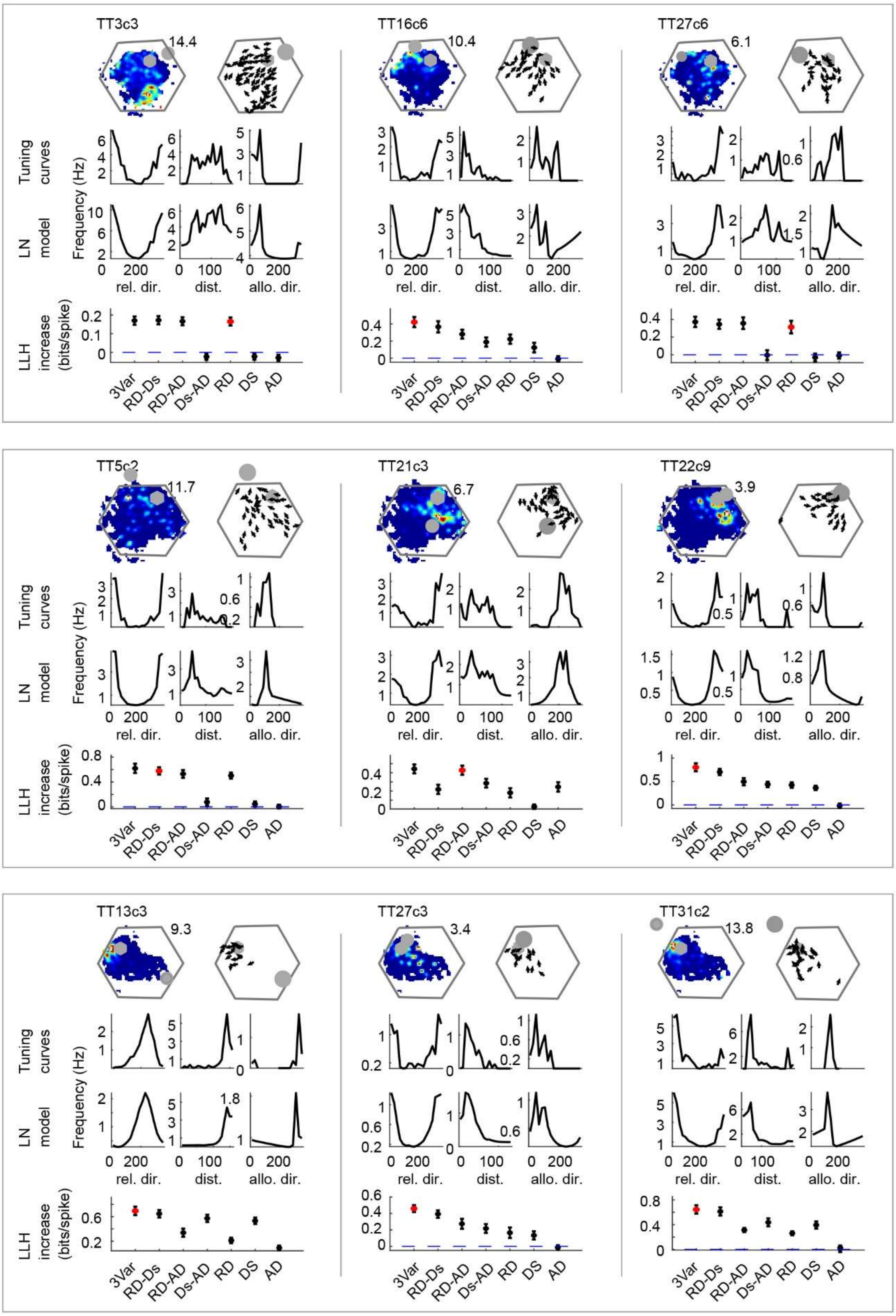

Rat 1
Rat 2
Rat 3
TT3c3
14.4
TT16c6
10.4
TT27c6
6.1
TT5c2
11.7
TT21c3
6.7
TT22c9
3.9
TT13c3
9.3
TT27c3
3.4
TT31c2
13.8
Tuning curves
LN model
Frequency (Hz)
rel. dir.
dist.
allo. dir.
LLH increase (bits/spike)
3Var
RD-Ds
RD-AD
Ds-AD
RD
DS
AD

Supplemental Figure 7 │ Representative examples of LN model results. The LN model analysis found that ConSink cells significantly encoded various combinations of relative direction, allocentric direction, and distance to the sink. Plots for individual cells show each cell's rate map (top left) with the goal (grey hexagon) and ConSink (grey closed circle) and the peak firing rate (to the top right of the rate map; the vector field (top right) showing spike-associated allocentric head direction by spatial position; tuning curves for relative direction, distance, and allocentric direction to the sink (second row, from left to right); the LN model response profiles (third row); the log likelihood increase in information about the firing rate for each nested LN model (3VAr indicates the model incorporating all 3 variables; RD, relative direction; Ds, distance; AD, allocentric direction). The significant model (i.e. the highest order model that produces a significant increase in information over the next lower-ordered model) is indicated in red (n = 10 repeats of the cross-validation procedure; error bars = s.e.m.).

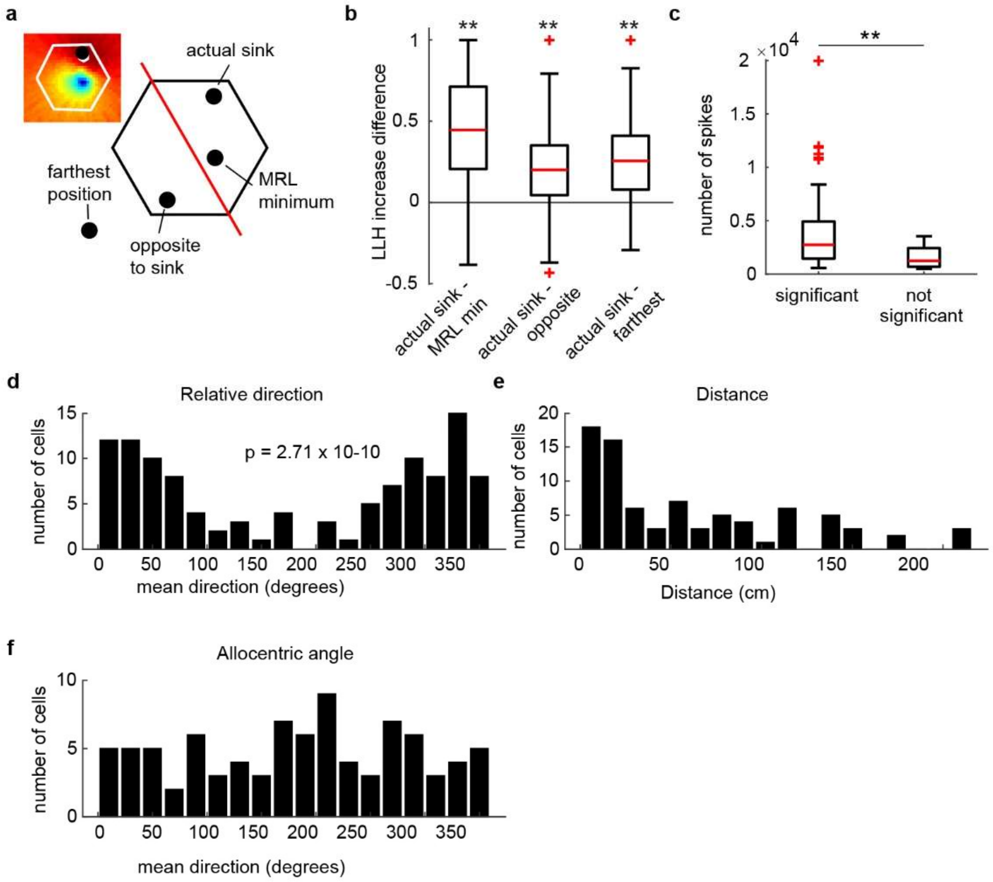

Supplemental Figure 8 │ Testing validity of LN model results. a, To test the validity of the LN model results, we recalculated the log-likelihood (LLH) increase in spike prediction using 3 non-sink control positions: first, we took the position of least tuning (i.e. MRL minimum; see inset, which is reproduced from Extended Data Fig. 5b, blue position), second, the sink position reflected across the x and y axes (opposite to sink), and last, the farthest distance in the field of view from the sink position. **b**, We calculated the difference between the LLH increase using the real sinks and using these 3 control sinks. The model provided significantly less information about a given ConSink cell's spiking when using any of these positions rather than the identified sink position itself (Wilcoxon signed-rank test, two-sided, n = 119 cells from 5 animals, z (from left to right) = 8.96, 7.75, 8.57; p (from left to right) = 3.38 x 10^{-19}, 9.49 x 10^{-15}, 1.08 x 10^{-17}). **c**, Cells not found significant by the

LN analysis fired fewer spikes than significant cells. Wilcoxon rank sum test, n = 123 significant cells, 19 not significant cells from 5 animals, $p = 3.39 \times 10^{-4}$. **d**, Relative direction, **e**, Distance, and **f**, Allocentric angle are well represented across the spectrum of possible values. Note that the circular distribution in (**d**) is significantly non-uniform ($p = 2.71 \times 10^{-10}$), as expected (see Fig. 1j). Plots show the population of peak values taken from the subset of ConSink cells found significant for the respective spatial variable. For box plots in (**b**) and (**c**), the central mark indicates the median, and the bottom and top edges of the box indicate the 25th and 75th percentiles, respectively; the whiskers extend to the most extreme data points within 1.5 times the interquartile range away from the bottom or top of the box, and all more extreme points are plotted individually using the '+' symbol.

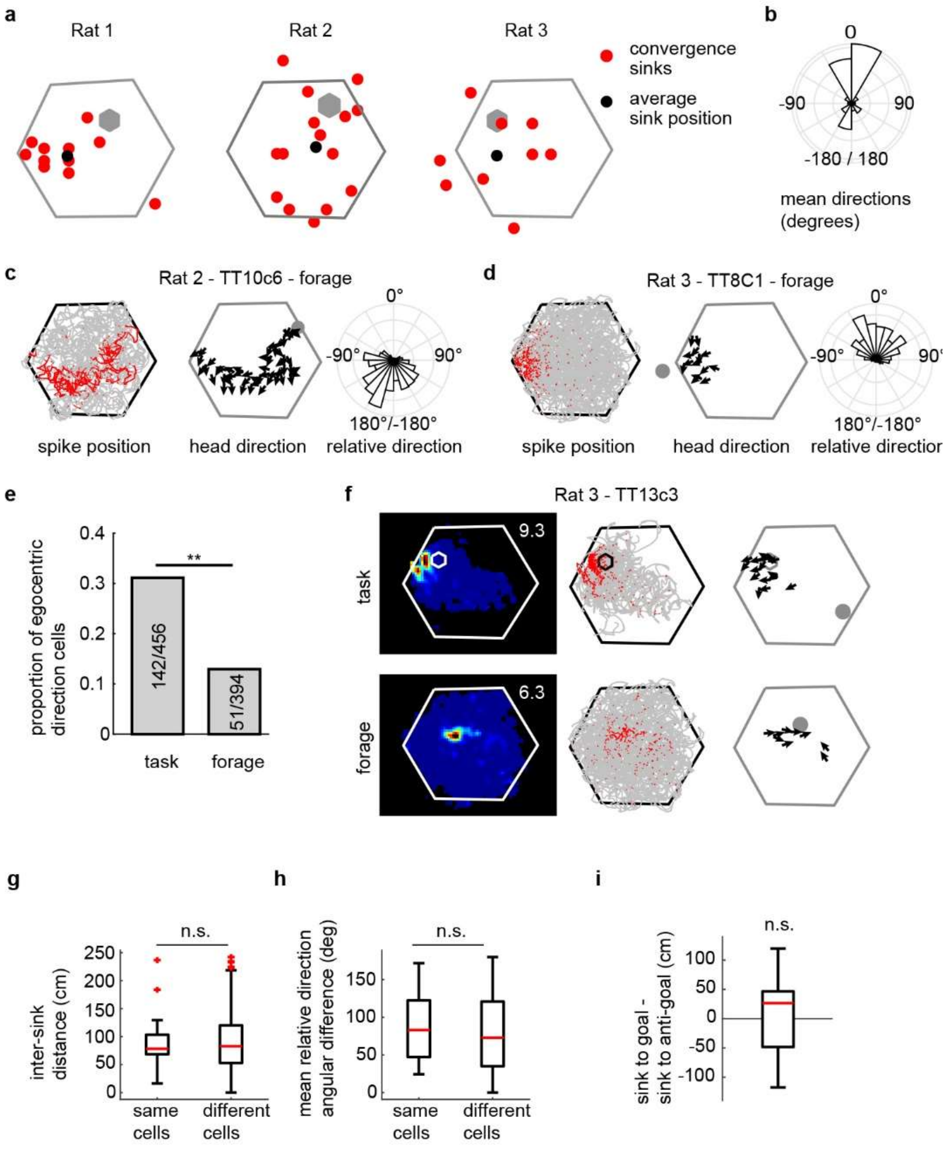

Supplemental Figure 9 │ ConSinks during open field foraging are fewer and not clustered. a, Distribution of ConSinks and average ConSink for animals 1 to 3. **b**, Average heading direction relative to the ConSink across all animals. **c**, **d**, Representative examples of ConSink cells during foraging. Left, paths (grey) and spikes (red). Middle, vector field depicts mean head direction at binned spatial positions; ConSink is shown as grey filled circle. Right, Polar plot showing the distribution of spike-associated head directions relative to the ConSink. **e**, More than twice as many place cells were significant for ConSink tuning during navigation

compared to foraging (Chi-square test, $X^2 = 39.9$, $p = 2.71 \times 10^{-10}$). **f**, Example of a Ca1 place cell with significant ConSinks during both navigation and foraging; note differences in field location, vector fields, and ConSink location. Peak firing rate is indicated at top right of rate map (left-most panel). **g**, The convergence sinks for the task and forage tasks were no closer within cells that were significantly modulated during both tasks than they were across cells significantly modulated during either tasks, indicating a reorganization between the two epochs (n = 13 (same cells), 1833 (different cells) from 5 animals, Wilcoxon rank sum test, two-sided, z = 0.081, p = 0.936). **h,** The same lack of a difference held for the preferred relative directions (n = 13 (same cells), 1833 (different cells) from 5 animals, Wilcoxon rank sun test, two-sided, z = 0.75, p = 0.452). **i,** ConSinks during foraging were no closer to the honeycomb task goals than to symmetrical locations on the opposite side of the maze (n = 51 cells from 5 animals, Wilcoxon signed rank test, two-sided, z = 0.52, p = 0.600; see Extended Data Fig. 6 for anti-goal positions). For box plots in (**g**-**i**), the central mark indicates the median, and the bottom and top edges of the box indicate the 25th and 75th percentiles, respectively; the whiskers extend to the most extreme data points within 1.5 times the interquartile range away from the bottom or top of the box, and all more extreme points are plotted individually using the '+' symbol.

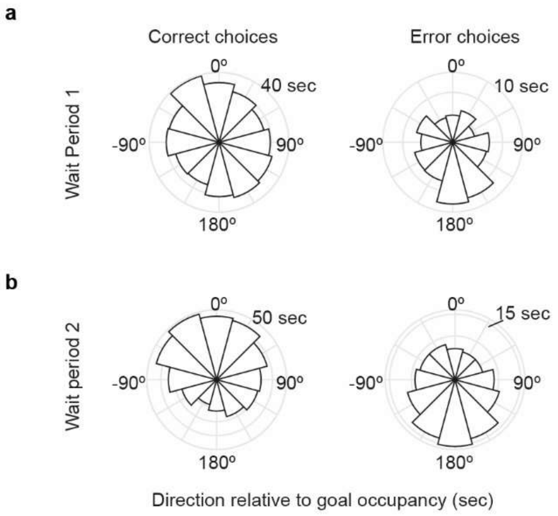

Supplemental Figure 10 │ Behavioural orientations during correct and error choices. a, The time spent oriented towards the goal in each possible orientation, averaged across the 3 animals, during Wait Period 1, which occurred after the previous platforms had lowered but before the next choice platforms were raised. Note the relatively uniform distribution during correct choices, while during errors, the animals orient away from goal (Rayleigh test for non-uniformity, Correct choices, z = 0.42 p = 0.66, Error choices, z = 2.59, p = 0.075). **b**, As in (**a**), but for Wait Period 2, which we define as the 4 seconds preceding the animals movement onto the Choice platform (Rayleigh test for non-uniformity, Correct choices, z = 14.23, p = 5.99×10^{-7}, Error choices, z = 4.42, p = 0.017).

Supplemental Video 1 │ Firing of neuron TT18c1 from Rat 2 during Trial 7 from Session 1. Note that the cell's convergence sink is shown as a red filled circle near the top of the screen. Spikes are indicated by smaller filled circles whose colour indicates relative firing rate (cold colours, lower; hot colours, higher). Firing rate is also indicated at lower right.
