# Supplementary material for: Hippocampal place cells have goal-oriented vector fields during navigation
Source: Nature. 2022 Jul 6;607(7920):741–6. doi: 10.1038/s41586-022-04913-9 (PMC9329099; doi:10.1038/s41586-022-04913-9)
Supplement: Supplementary file 2 — Reporting Summary [file 41586_2022_4913_MOESM2_ESM.pdf]

nature portfolio

Corresponding author(s): J. Ormond

Last updated by author(s): Apr 7, 2022

Reporting Summary

Nature Portfolio wishes to improve the reproducibility of the work that we publish. This form provides structure for consistency and transparency in reporting. For further information on Nature Portfolio policies, see our Editorial Policies and the Editorial Policy Checklist.

Statistics

For all statistical analyses, confirm that the following items are present in the figure legend, table legend, main text, or Methods section.

n/a	Confirmed	
☐	☒	The exact sample size (*n*) for each experimental group/condition, given as a discrete number and unit of measurement
☒	☐	A statement on whether measurements were taken from distinct samples or whether the same sample was measured repeatedly
☐	☒	The statistical test(s) used AND whether they are one- or two-sided<br>*Only common tests should be described solely by name; describe more complex techniques in the Methods section.*
☒	☐	A description of all covariates tested
☒	☐	A description of any assumptions or corrections, such as tests of normality and adjustment for multiple comparisons
☐	☒	A full description of the statistical parameters including central tendency (e.g. means) or other basic estimates (e.g. regression coefficient) AND variation (e.g. standard deviation) or associated estimates of uncertainty (e.g. confidence intervals)
☐	☒	For null hypothesis testing, the test statistic (e.g. *F*, *t*, *r*) with confidence intervals, effect sizes, degrees of freedom and *P* value noted<br>*Give P values as exact values whenever suitable.*
☒	☐	For Bayesian analysis, information on the choice of priors and Markov chain Monte Carlo settings
☒	☐	For hierarchical and complex designs, identification of the appropriate level for tests and full reporting of outcomes
☐	☒	Estimates of effect sizes (e.g. Cohen's *d*, Pearson's *r*), indicating how they were calculated

Our web collection on statistics for biologists contains articles on many of the points above.

Software and code

Policy information about availability of computer code

Data collection	Custom code written in LabView 2018 was used to collect video data. Eletrophysiology data was collected using commercial software (Intan RHD data acquisition software Version 1.5.2).
Data analysis	Data analysis used custom code written in Matlab 2019a. Spike sorting performed using KiloSort (version 1) and Phy 2.0. Animal tracking performed in DeepLabCut2.0.

For manuscripts utilizing custom algorithms or software that are central to the research but not yet described in published literature, software must be made available to editors and reviewers. We strongly encourage code deposition in a community repository (e.g. GitHub). See the Nature Portfolio guidelines for submitting code & software for further information.

Data

Policy information about availability of data

All manuscripts must include a data availability statement. This statement should provide the following information, where applicable:

- Accession codes, unique identifiers, or web links for publicly available datasets
- A description of any restrictions on data availability
- For clinical datasets or third party data, please ensure that the statement adheres to our policy

The datasets generated during the current study are available from the corresponding authors on reasonable request. Source data are provided with this paper.

Field-specific reporting

Please select the one below that is the best fit for your research. If you are not sure, read the appropriate sections before making your selection.

☒ Life sciences ☐ Behavioural & social sciences ☐ Ecological, evolutionary & environmental sciences

For a reference copy of the document with all sections, see nature.com/documents/nr-reporting-summary-flat.pdf

Life sciences study design

All studies must disclose on these points even when the disclosure is negative.

Sample size	No statistical methods were used to pre-determine sample size. Numbers of animals used were based on those used in the authors' previous publications as well as other studies in the same field (e.g. Ormond and McNaughton, PNAS, 2015; Wang, Foster, and Pfeiffer et al., Science, 2020).
Data exclusions	No data were excluded.
Replication	ConSink cells were identified in all 5 animals used in the study. The findings reported in Figures 1-3 (and associated Extended Data figures) were presented for each individual animal (in addition to grouped population analyses).
Randomization	Our study did not use separate groups, so randomization was not used.
Blinding	Our study did not use separate groups, so blinding was not used.

Reporting for specific materials, systems and methods

We require information from authors about some types of materials, experimental systems and methods used in many studies. Here, indicate whether each material, system or method listed is relevant to your study. If you are not sure if a list item applies to your research, read the appropriate section before selecting a response.

Materials & experimental systems

n/a	Involved in the study	
☒	☐	Antibodies
☒	☐	Eukaryotic cell lines
☒	☐	Palaeontology and archaeology
☐	☒	Animals and other organisms
☒	☐	Human research participants
☒	☐	Clinical data
☒	☐	Dual use research of concern

Methods

n/a	Involved in the study	
☒	☐	ChIP-seq
☒	☐	Flow cytometry
☒	☐	MRI-based neuroimaging

Animals and other organisms

Policy information about studies involving animals; ARRIVE guidelines recommended for reporting animal research

Laboratory animals	5 male Lister Hooded rats, aged 9-12 months, purchased from Charles River Laboratories.
Wild animals	n/a
Field-collected samples	n/a
Ethics oversight	Experiments were carried out in accordance with British Home Office Regulations, and study protocols were reviewed by the Animal Welfare and Ethical Review Board at University College London.

Note that full information on the approval of the study protocol must also be provided in the manuscript.

March 2021
